# AI in transplantation: levelling the playing field and raising the methodological standards

**DOI:** 10.3389/ti.2026.17015

**Published:** 2026-07-14

**Authors:** Nina Pilat, Maria Irene Bellini, Thierry Berney, Olivier Aubert

**Affiliations:** 1 Deputy Editor-in-Chief, Transplant International; 2 Department of Cardiac and Thoracic Aortic Surgery, Medical University of Vienna, Vienna, Austria; 3 Center for Biomedical Research and Translational Surgery, Medical University of Vienna, Vienna, Austria; 4 Department of Surgery, Sapienza University, Rome, Italy; 5 Editor-in-Chief, Transplant International; 6 Division of Transplantation, Immunology and Nephrology, Hospices Civils de Lyon, Lyon, France; 7 Executive Editor, Transplant International; 8 Department of Kidney and Metabolic Diseases, Transplantation and Clinical Immunology, Necker Hospital, Assistance Publique - Hôpitaux de Paris, Paris, France; 9 Université Paris Cité, Institut National de la Santé et de la Recherche Médicale U1151, Centre National de la Recherche Scientifique Unités Mixtes de Recherche 8253, Institut Necker Enfants Malades, Paris, France

**Keywords:** artificial intelligence (AI), methodological standards, research equity, scientific publishing, transplantation medicine

## Introduction

Artificial intelligence has rapidly entered the scientific publishing ecosystem, often faster than the norms designed to govern it. A recent analysis of more than 1.1 million papers across arXiv, bioRxiv, and Nature portfolio journals estimates that text modified by large language models has been rising steadily since early 2023, reaching up to 22% of abstracts in computer science and roughly 9% in the life sciences [1]. This shift is broad, global, and most pronounced among authors from regions where English is not the dominant language. Importantly, the transformation extends well beyond writing. AI is increasingly shaping how we generate hypotheses, build predictive models, analyze imaging and omics data, and interpret what counts as evidence. As editors in transplantation medicine, we believe that both fronts, linguistic and methodological, require the same editorial response: not rejection of the technology, but the construction of standards that allow its responsible use.

## Levelling the linguistic field

For decades, science has been built on an implicit assumption that fluent English reflects good scholarship, and that linguistic proficiency signals intellectual merit. The consequences have been well documented. Non-native English speakers spend disproportionate time, financial resources, and effort to produce manuscripts that appears review-ready, while native speakers benefit from an invisible advantage. The manifold costs of conducting science in a second or third language, ranging from delays in publication to reduced citation visibility and limited participation in international debates have been extensively documented [2]. Of note, GPT-detection tools have been shown to systematically misclassify non-native writing as machine-generated with error rates above 60%, while achieving near-perfect accuracy on samples written by native English speakers [3]. When detection tools themselves carry such language bias, any attempt to police AI use rests on an unstable basis.

Against that backdrop, AI can act as a meaningful equalizer. Nature Methods has explicitly acknowledged that generative models can help scientists who struggle with language or writing to refine their manuscripts and communicate more clearly [4]. Elsevier, Springer Nature, and other major publishers now allow AI-assisted editing, provided that authors remain accountable for content and disclose their use. In practice, disclosure remains limited: only 2 out of 200 randomly sampled computer science papers reported the use of large language models in writing [1]. Taken together, these data suggest that AI is quietly smoothing the edges of scholarly English across disciplines, and the relevant question is no longer whether this is happening, but under which conditions it should be accepted.

This raises an uncomfortable but necessary question: if a manuscript becomes easier to read because an author used AI to improve their English, does that diminish the underlying science, or does it allow reviewers to judge research more directly on its substance? The academic community has long conflated laborious writing with intellectual rigor, although no elegant prose can rescue a weak hypothesis, and no awkward syntax negates a well-designed study. AI may help bring this conflation into focus [5]. The effect is particularly relevant for early-career researchers, for whom the hidden curriculum of stylistic fluency often weighs as heavily as the science itself. Used responsibly, AI can act as a structural scaffold that helps younger researchers organize their ideas and find their professional voice, provided that they remain the authors of what they publish. For decades, young scientists have been socialised to mimic their supervisors’ writing style, as though acquiring the “right” academic voice were a prerequisite for belonging to the ivory tower. But elegant phrasing is not the essence of science and if AI can take over some of the stylistic tasks, it may finally allow early-career researchers to invest more of their limited time where it belongs: in designing better studies, interrogating data more critically and nurturing genuine scientific curiosity.

Notwithstanding these potential benefits, the risks should not be underestimated. The Nature Methods guidance highlights accuracy, hallucination, and unverifiable claims as persistent concerns, requiring active human oversight and explicit responsibility [4]. Large-scale corpus analyses have also reported that heavy LLM use correlates with greater stylistic homogeneity across papers, an early signal that overreliance could flatten scholarly voice and reduce diversity of expression [1]. In peer review, uploading manuscripts into external AI platforms compromises confidentiality, a breach now explicitly prohibited by most publishers. The more ambitious vision of a multilingual publishing ecosystem, in which authors write in their own language and reviewers receive AI-translated versions, remains aspirational and raises additional equity concerns related to translation quality for low-resource languages [6]. These risks do not justify rejection of the technology. These argue for clear standards.

## Beyond language: AI as methods

If AI is reshaping how we write about science, it is reshaping even more profoundly how we conduct it. Predictive models of graft survival, deep learning classifiers of allograft biopsies, transcriptomic signatures of rejection, foundation models trained on multimodal clinical data, and conversational agents embedded in clinical workflows are no longer prospective developments. They are already present in our literature, in our practice, and increasingly in our editorial submissions. Here too, an inequity is at work, although of a different kind. It is no longer primarily a linguistic gap, but a methodological one.

The technical barrier to building models has fallen sharply. Open-source machine learning libraries, accessible tutorials, and large language models capable of generating analytical pipelines on request have made it possible to produce a predictive model in a weekend. The conceptual barrier to understanding what these models actually do, and which claims they can legitimately support, has not decreased; if anything, it has increased. The same literature that helps levelling the writing field also produces papers in which data leakage is unrecognized, calibration is not reported, external validation is missing, and predictive performance is conflated with causal effect. These shortcomings are not exotic. They are systemic patterns documented across the AI-in-medicine literature, and transplantation is no exception [7, 8].

This second front matters because the consequences differ. A poorly written but methodologically sound paper represents a missed opportunity for the reader. A fluently written but methodologically unsound AI paper may lead to a misallocation of clinical attention, and, if the model is ultimately deployed, of patient care. The editorial response to AI in transplantation cannot stop at the level of the manuscript’s surface. It should also engage with the methods themselves and the quality of the evidence they produce.

## What this calls for editorially

To address this dual responsibility, Transplant International is launching a new editorial series entitled Decoding AI for Transplantation. The series is designed to fill the gap that the linguistic discussion alone cannot cover. Across recurring issues, it will provide short and structured primers, each centered on a single methodological or implementation concept, illustrated with a clinical scenario, and closing with a set of actionable questions that readers, reviewers, and clinical users can apply when evaluating an AI tool. Topics will span the full path from manuscript to bedside, including calibration and decision curve analysis, data leakage in longitudinal transplant data, external validation and transportability, the distinction between prediction and causal inference, uncertainty quantification, survival analysis with deep learning, digital pathology of allograft biopsies, the critical appraisal of foundation models and large language models, the integration of AI tools into transplant clinical workflows, post-deployment monitoring and performance drift, and the assessment of AI tools as clinical interventions in their own right.

This breadth reflects a deliberate editorial choice. The questions raised by AI in transplantation cannot be answered solely at the level of the published manuscript. Predictive models, image classifiers, and decision support tools are increasingly proposed for use in clinical practice, often before their behaviour in routine workflows is well characterized. Of note, the introduction of an AI tool is itself a form of clinical intervention, with potential effects on biopsy thresholds, treatment decisions, alert handling, and patient outcomes. These effects deserve the same scrutiny applied to any other intervention in transplantation. Issues such as how performance should be monitored over time and across centers, how model outputs should be communicated to clinicians and patients, how integration into electronic health records can avoid alert fatigue, and how equitable access can be preserved across centers with heterogeneous resources are central to the responsible use of AI in our field, and will be addressed alongside the methodological primers.

The objective is not to celebrate AI, nor to catalogue its applications. It is to equip the transplantation community with the conceptual tools required to read AI papers carefully, to review them rigorously, to assess their suitability for clinical deployment, to monitor their performance once in use, and, when authors themselves, to design and report their work in ways that will withstand scrutiny. Each primer will be written by invited teams combining clinical, methodological, and implementation expertise, with a recognizable structure and a unified visual style across issues. The intent is to maintain a didactic tone, a critical perspective, and an honest discussion of the limitations of the methods and tools presented. At its core, AI is a powerful engine for pattern recognition and imitation and superior in copying, summarising, and amplifying, but it does not originate purpose or curiosity. Innovation in science still begins with a human mind willing to ask a question that has never been asked before, reative leap cannot simply be automated.

Taken together, levelling the linguistic field and raising the methodological standards are not competing aims. They reflect a single editorial commitment. AI will not make weak studies strong, and it will not, on its own, make our literature more equitable or more rigorous. However, it has made the costs of inaction more visible than they were a decade ago. The task of editors in transplantation is to build the norms that allow responsible use of AI in writing, in reviewing, and in the methods on which our claims ultimately rely, so that scientific merit can be assessed on the basis of the science itself.

## References

[1] Liang W Zhang Y Wu Z Lepp H Ji W Zhao X Quantifying large language model usage in scientific papers. Nat Hum Behav (2025) 9(12):2599–609. 10.1038/s41562-025-02273-8 40760036

[2] Amano T Ramirez-Castaneda V Berdejo-Espinola V Borokini I Chowdhury S Golivets M The manifold costs of being a non-native English speaker in science. PLoS Biol (2023) 21(7):e3002184. 10.1371/journal.pbio.3002184 37463136 PMC10353817

[3] Liang W Yuksekgonul M Mao Y Wu E Zou J . GPT detectors are biased against non-native English writers. Patterns (N Y) (2023) 4(7):100779. 10.1016/j.patter.2023.100779 37521038 PMC10382961

[4] Using AI responsibly in scientific publishing. Nat Methods (2026) 23(2):271. 10.1038/s41592-026-03020-1 41673429

[5] Berdejo-Espinola V Amano T . AI tools can improve equity in science. Science (2023) 379(6636):991. 10.1126/science.adg9714 36893248

[6] Amano T Bowker L Burton-Jones A . AI-mediated translation presents two possible futures for academic publishing in a multilingual world. PLoS Biol (2025) 23(6):e3003215. 10.1371/journal.pbio.3003215 40549671 PMC12185007

[7] Collins GS Moons KGM Dhiman P Riley RD Beam AL Van Calster B TRIPOD+AI statement: updated guidance for reporting clinical prediction models that use regression or machine learning methods. BMJ (2024) 385:e078378. 10.1136/bmj-2023-078378 38626948 PMC11019967

[8] Van Calster B McLernon DJ van Smeden M Wynants L Steyerberg EW Topic Group 'Evaluating diagnostic t Calibration: the achilles heel of predictive analytics. BMC Med (2019) 17(1):230. 10.1186/s12916-019-1466-7 31842878 PMC6912996

